# TCR Repertoire Characterization for T Cells Expanded in Response to hRSV Infection in Mice Immunized with a Recombinant BCG Vaccine

**DOI:** 10.3390/v12020233

**Published:** 2020-02-20

**Authors:** Emma Rey-Jurado, Karen Bohmwald, Hernán G. Correa, Alexis M. Kalergis

**Affiliations:** 1 Millennium Institute on Immunology and Immunotherapy, Departamento de Genética Molecular y Microbiología, Facultad de Ciencias Biológicas, Pontificia Universidad Católica de Chile, Santiago 8331010, Chile; emmareyjurado@gmail.com (E.R.-J.); kbohmwald@uc.cl (K.B.); nanocorrea.ua@gmail.com (H.G.C.); 2 Departamento de Endocrinología, Escuela de Medicina, Facultad de Medicina, Pontificia Universidad Católica de Chile, Santiago 8331010, Chile

**Keywords:** human respiratory syncytial virus, recombinant BCG vaccine, TCR repertoire

## Abstract

T cells play an essential role in the immune response against the human respiratory syncytial virus (hRSV). It has been described that both CD4^+^ and CD8^+^ T cells can contribute to the clearance of the virus during an infection. However, for some individuals, such an immune response can lead to an exacerbated and detrimental inflammatory response with high recruitment of neutrophils to the lungs. The receptor of most T cells is a heterodimer consisting of α and β chains (αβTCR) that upon antigen engagement induces the activation of these cells. The αβTCR molecule displays a broad sequence diversity that defines the T cell repertoire of an individual. In our laboratory, a recombinant Bacille Calmette–Guérin (BCG) vaccine expressing the nucleoprotein (N) of hRSV (rBCG-N-hRSV) was developed. Such a vaccine induces T cells with a Th1 polarized phenotype that promote the clearance of hRSV infection without causing inflammatory lung damage. Importantly, as part of this work, the T cell receptor (TCR) repertoire of T cells expanded after hRSV infection in naïve and rBCG-N-hRSV-immunized mice was characterized. A more diverse TCR repertoire was observed in the lungs from rBCG-N-hRSV-immunized as compared to unimmunized hRSV-infected mice, suggesting that vaccination with the recombinant rBCG-N-hRSV vaccine triggers the expansion of T cell populations that recognize more viral epitopes. Furthermore, differential expansion of certain TCRVβ chains was found for hRSV infection (TCRVβ^+^8.3 and TCRVβ^+^5.1,5.2) as compared to rBCG-N-hRSV vaccination (TCRVβ^+^11 and TCRVβ^+^12). Our findings contribute to better understanding the T cell response during hRSV infection, as well as the functioning of a vaccine that induces a protective T cell immunity against this virus.

## 1. Introduction

The human respiratory syncytial virus (hRSV) is a major cause of acute tract respiratory infections (ALRTI) in children and the elderly [1]. Infection with hRSV produces pulmonary inflammatory hyperresponsiveness with significant lung damage due to a T helper 2 (Th2)-biased immune response in the susceptible [2]. Along these lines, elevated Th2 cytokines including IL-3, IL-4, IL-10 and IL-13 were found in the airways in infants with hRSV bronchiolitis [3]. Upon hRSV infection in mice, moderate bronchiolitis, reduced activity and weight loss is observed [4,5]. Also, hRSV-associated immunopathology can be measured by body weight loss and infiltration of neutrophils into the lungs [6]. Further, CD4^+^ and CD8^+^ T cells are important for the immune response against hRSV infection [7]. Although several vaccine approaches have been evaluated, up to date, no licensed vaccine is available. To prevent hRSV-induced disease, we have developed a recombinant Bacille Calmette–Guérin (BCG) expressing the nucleoprotein (N) of hRSV (rBCG-N-hRSV), which induces the expansion of T cells with a Th1-polarized phenotype that promotes viral clearance without causing inflammatory lung damage [8,9]. We have shown that both CD4^+^ and CD8^+^ T cells contribute to the clearance of hRSV after immunization with rBCG-N-hRSV. The receptor of T cells (TCR) consists of a heterodimeric protein composed of α and β chains linked by a disulfide bond and associated with an array of accessory signaling proteins, known as the CD3 complex [10]. The TCR α and β chains contain constant and a variable (V) domains [11]. During T cell development in the thymus, Vα and Vβ gene segments undergo genetic recombination events that lead to a broad diversity of TCR sequences, which constitute the TCR repertoire [11,12]. Further, it has been estimated that after thymic selection, the diversity TCRαβ receptors can reach up to 10^7^ TCRs circulating in the periphery at a given time of the individual life [11]. During a viral infection, antigen-presenting cells (APC) uptake and process proteins derived from pathogens, which then will be presented to T cells as small peptides bound to MHC molecules [13]. Because the number of viral epitopes is limited, APCs present epitopes in a hierarchical manner to T cells [11]. During an hRSV infection, CD4^+^ and CD8^+^ T cells responses focus mostly on a few epitopes derived from the F, G, M2, P and N proteins [14,15,16,17].

A differential expansion of various elements of the TCR repertoire has been observed after infection by viruses, such as hepatitis virus C, human papilloma virus, Epstein–Barr virus, and influenza A [18,19,20,21]. Interestingly, mice vaccinated with purified hRSV-G protein and challenged with hRSV showed a significant expansion of TCRVβ14^+^ CD4^+^ T cells that infiltrate the lungs, with a subsequent increase of the airway immunopathology [22]. Further, the depletion of TCRVβ14^+^ CD4^+^ T cells in rats led to a decrease of eosinophil infiltration into the lungs and prevented the Th2-biased immune response [22]. These results underscore the role of the TCR repertoire not only for the activation and proliferation of T cells but also for driving to a favorable response after an antigen exposure. Furthermore, M2_82-90_-specific T cells were shown to almost exclusively use TCRVβ13 and TCRVβ8.1-8.2 in CB6F1 and BALB/c mice, respectively [23,24]. Upon vaccination with rBCG-N-hRSV and infection with hRSV, protective early recruitment of hRSV-specific T cells were found in the lungs of mice [8]. Such hRSV-specific T cells are responsible for preventing hRSV infection and could display a TCR repertoire similar to uninfected healthy mice [6]. However, since repeated antigen exposure occurred in those animals, the TCR repertoire could become even more restrictive [25]. Such a phenomenon has also been found after persistent infections, in which exposure to antigen is continuous over time [19].

In the present study, we have characterized the TCR repertoire of T cells expanded in response to hRSV infection both in naïve and rBCG-N-hRSV immunized mice. Significant differences in TCRVβ repertoire usage were observed between unimmunized and rBCG-N-hRSV-immunized mice, upon hRSV infection. Importantly, a more diverse TCR repertoire in CD4^+^ T cells was found in the lungs from rBCG-N-hRSV-immunized mice as compared with naïve animals, herein revealing that vaccination might promote a T cell response to a diversity of hRSV antigens. Thereby, vaccination with rBCG-N-hRSV not only prevents from hRSV infection but also triggers a favorable expansion of T cells that prevents the disease caused by this virus.

Furthermore, while we found an increased frequency for TCRVβ^+^8.3 and TCRVβ^+^5.1,5.2 in hRSV-infected-mice, TCRVβ^+^11 and the TCRVβ^+^12 preferentially expanded after rBCG-N-hRSV vaccination. Also, hRSV-specific T cells expanded from the spleens of virus-infected mice displayed a more diverse TCR repertoire as compared to T cell within the lungs. Differences in the TCR repertoire between spleens and lungs suggest that populations of CD4^+^ and CD8^+^ T cells vary from the spleen to the site of infection of hRSV (lungs). Our findings contribute to a better understanding of the T cell response against hRSV infection in the context of natural hRSV infection and immunization with a rBCG vaccine.

## 2. Materials and Methods

### 2.1. Viruses

HEp-2 monolayers were grown in 10% FBS-DMEM up to 70%–80% confluence that cells were inoculated with hRSV at a multiplicity of infection (MOI) equal to one (strain 13018–8) [9] in 1% FBS-DMEM. After 2 h, the medium was changed, and cells were cultured for 72 h at 37 °C until cytopathic effect was observed. During virus collection, cells were scrapped, and the infectious medium was pooled and centrifuged at 500× *g* for 5 min to remove cell debris. In parallel, supernatants of noninfected monolayers cells were collected and used as noninfectious control (mock) for each the virus preparations, as previously described [9].

### 2.2. Animals

BALB/cJ mice were originally obtained from the Jackson Laboratory (Bar Harbor, ME). Animals were maintained at the pathogen-free animal facility at the Pontificia Universidad Católica de Chile (Santiago, Chile). All animal work was performed according to institutional guidelines and supervised by a trained veterinarian, which was approved by the Animal Ethics Committee of the Pontificia Universidad Católica de Chile (protocol number 150505003).

### 2.3. hRSV Infection and rBCG-N-hRSV Immunization

Six- to eight-week-old male BALB/cJ mice were immunized with subcutaneous injection in the right dorsal flank with 1 × 10^8^ CFU rBCG strains expressing the N-hRSV protein in a final volume of 100 μL per dose. Vaccine doses were prepared as previously described [26]. At day 14 postimmunization mice were boosted with the rBCG-N-hRSV and at day 21 challenged intranasally (i.n.) with 1 × 10^6^ PFU of hRSV. Disease parameters, such as body weight, were determined daily after infection. Four days after infection, mice were terminally anesthetized by i.p. injection of a mixture of ketamine and xylazine (100 and 5 mg/kg, respectively). For a set of experiments with mock-treated and hRSV-infected mice, mice were euthanized seven days postinfection. The left lung was occluded using Kelly hemostatic forceps and bronchoalveolar lavage fluid (BALF) was obtained by gently instilling intratracheally 1 mL of 5% FBS-PBS three times. Spleens and lungs were collected. After BALF collection, the right lung was cut into small pieces and incubated for 1 h at 37 °C in PBS containing 1 mg/mL of collagenase type IV (Thermo Fisher Scientific, Catalog No. 17104-019) and 50 mg/mL of DNase. After ammonium-chloride-potassium (ACK) treatment and 1× PBS washes, cells were processed for flow cytometry staining. Single cell suspensions derived from spleens were stimulated with heat-inactivated hRSV (HI-hRSV) at a final multiplicity of infection (MOI) equal to five plaque-forming units (PFUs/cell) to expand hRSV-specific T cells. CD3/CD28-treated and unstimulated cells, as positive and negative controls, respectively, were included. Three days later, cells were recovered, washed and processed for flow cytometry analyses. Supernatants were collected and IFN-γ secretion was measured by ELISA (BD OptEIA™). To perform histopathology analyses, the left bronchus of the left lung was clamped using Kelly hemostatic forceps. After obtaining the BALF of the right lung, the left lung was fixed with 4% paraformaldehyde. The lung was embedded, sliced and stained for histopathology analyses using hematoxylin and eosin (HE), as previously described [9]. The rest of the lung was collected and incubated with collagenase IV for 30 min at 37 °C, and homogenized with PBS-1X/1%FBS using a 70 μm cell strainer (BD Biosciences) and centrifuged at 0.3 *g* for 5 min at 4 °C. For some experiments, T cells were purified from lungs using Percoll gradient followed by MACS T cell isolation kits (Miltenyi Biotec). Subsequently, T cells purified from three lungs of same experimental group were pooled to obtain sufficient cells for the analyses. Bone marrow-derived dendritic cells (DCs) from BALB/cJ mice were prepared as described previously [27]. Briefly, DCs were grown in RPMI 1640 medium with 10% FBS and supplemented with murine GM-CSF. On day 5 of culture, DCs were pulsed with heat-inactivated hRSV (HI-hRSV) at a final multiplicity of infection (MOI) equal to five plaque-forming units (PFUs/cell). Afterward, hRSV-pulsed DCs were cocultured with purified T cells from the lungs at a ratio equal to 1:1.

### 2.4. Flow Cytometry Analyses

To analyze the infiltration of inflammatory cells in BALF, cells were processed for flow cytometry (FACS) staining using the following antibodies: anti-Ly-6G FITC (clone 1A8), anti-Siglec-F PE (clone ESO-2440), anti-CD11c PeCy7, anti-CD11b APCCy7, anti-I/A-I/E (MHC-II) BV605, CD45 BV786. To analyze the T cell activation after expansion with hRSV-pulsed DCs, cells were stained with CD69 PE and TCR APC. To analyze the TCRVβ repertoire in the lungs and spleens, cells were stained using CD45 BV786, CD4 Percp 5.5, CD8 APCCy7, and the following antibodies panels for each sample: 1) TCRVβ12 (clone MR11-1) FITC, TCRVβ3.1 (clone KJ25) PE and TCRVβ11 (clone KT11) APC; 2) TCRVβ13 (clone MR12-3) FITC, TCRVβ4 (clone KT4) PE and TCRVβ8.1 (clone KJ16-133) APC; 3) TCRVβ17 (clone KJ23) FITC, TCRVβ10 (clone B21.5) PE and TCRVβ5.1,5.2 (clone MR9-4) APC; 4) TCRVβ14 (clone 14-2) FITC, TCRVβ8.3 (clone 1B3.3) PE and TCRVβ6 (clone RR4-7) APC; 5) TCRVβ8 (clone MR5-2) FITC, TCRVβ7 (clone TR310) PE and TCRVβ2 (clone B206) APC; 6) TCRVβ9 (clone MR10-2) FITC. Absolute cell counts were determined using CountBright^TM^ absolute counting beads in a 1:30 dilution in a maximum volume of 100 μL and acquired in a FACS BD LSR FORTESSA X-20 flow cytometer (BD Biosciences). Data were analyzed using FlowJo v X 10.0.7 (FlowJo, LLC) (Figure S1). As the absolute number of T cells with a certain TCRVβ is relevant, we evaluated the absolute number of TCRVβ^+^ CD4^+^ or CD8^+^ T cells. Also, as rBCG-N-hRSV immunization induces an early T cell recruitment [6], the total count of all TCRVβ^+^ evaluated was included for the TCR repertoire analysis. Then, to obtain the frequency of a specific TCRVβ chain, the following formula was used: (TCRVβ^+^ CD4^+^ or CD8^+^ T cell count/Sum of TCRVβ^+^ evaluated CD4^+^ or CD8^+^ T cell count) × 100.

### 2.5. Statistical Analyses

All statistical analyses were performed using GraphPad Prism 5.0 (GraphPad Software). Statistical significance was assessed using one-way ANOVA, two-way ANOVA and *t*-students tests. Differences were considered significant with a *p*-value < 0.05. The frequency of TCRVβ^+^ was compared among unimmunized-mock treated, unimmunized-hRSV-infected, and rBCG-N-hRSV-immunized-hRSV-infected mice. 

## 3. Results

### 3.1. TCR Repertoire of T Cells from hRSV-Infected Mice

**Figure 1:**
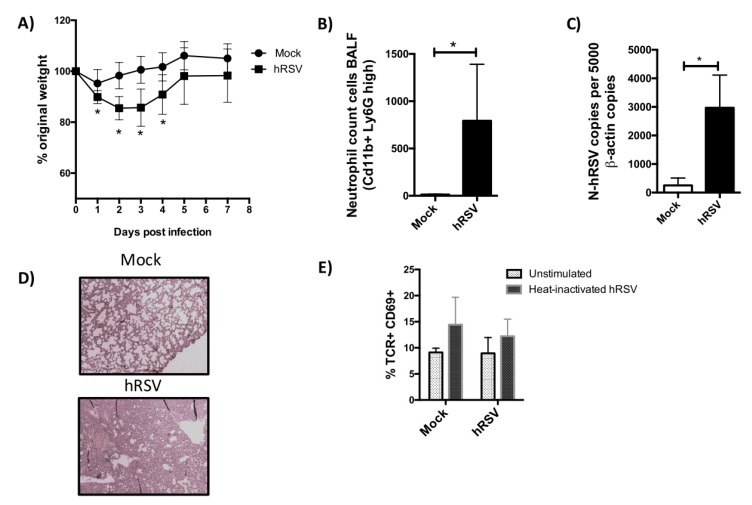
T cell expansion induced by human respiratory syncytial virus (hRSV) infection. BALB/cJ mice were infected with 1 × 10^6^ PFU of hRSV and 7 days after infection, mice were euthanized. Bodyweight was measured for seven days (**A**). Neutrophils in the BALF were evaluated by FACS (**B**). Lung damage was evaluated by lung histology (**C**). Viral loads were evaluated by RT-qPCR (**D**). Lung % TCR^+^ CD69^+^ cells (**E**). The spleens of mice sacrificed at seventh day post-infection were collected, and the resulting splenocytes were then stimulated with heat-inactivated hRSV (HI-hRSV) (57 °C for 30 min). Seventy-two hours poststimulation the TCR^+^ CD69^+^ cells as a T cell activation marker were detected by flow cytometry. (**A**) Two-way ANOVA paired test and multiple *t*-students test were performed to assess statistical differences (* *p* < 0.05) indicating statistical difference in (i) time and infection condition, (ii) significant decrease of percent original weight in hRSV-infected as compared to mock-treated mice at days 1, 2, 3 and 4 postinfection. (**A**,**D**,**E**) *t*-students tests were performed (* *p* < 0.05). Bars represent mean ± SD in all graphs.

To evaluate the TCR repertoire of T cells in response to hRSV infection, BALB/cJ mice were infected with 1 × 10^6^ PFU of hRSV. Disease parameters, such as body weight, neutrophils infiltration to the lungs and viral load, were determined after infection. As expected, a significant loss of original weight was shown by hRSV-infected mice (Figure 1A). As previously reported, the recruitment of neutrophils into the lungs and airways viral loads were observed for hRSV-infected mice (Figure 1B,C). Furthermore, infiltration of inflammatory cells was observed in histological analyses of lung samples from hRSV-infected, as compared to mock-treated mice (Figure 1D). The expansion of T cells by hRSV-loaded DCs from the spleens of hRSV-infected mice was determined by measuring activated T cells (TCR^+^CD69^+^) by flow cytometry (Figure 1E). Both mock-treated and hRSV-infected cocultures showed a slight increase of CD69 expression after stimulation with heat-inactivated hRSV, herein indicating that the response was unspecific to the stimulus. 

**Figure 2:**
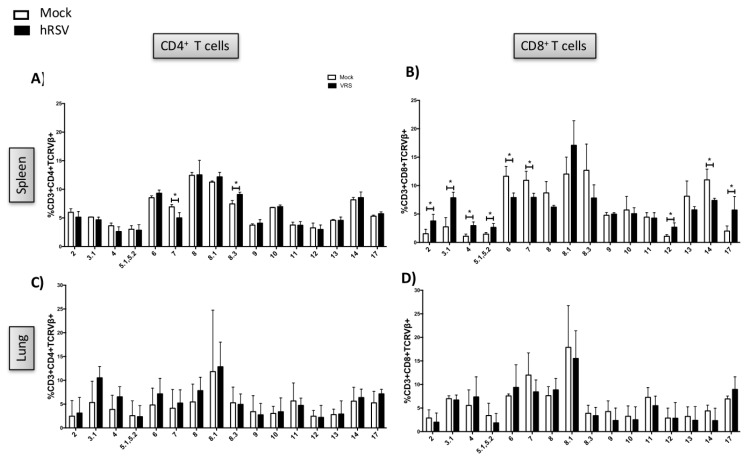
TCR repertoire of T cells expanded by hRSV infection. BALB/cJ mice were infected with 1 × 106 PFU of hRSV and 7 days after infection mice were euthanized. To determine the repertoire of the TCR in the lungs, purified T cells from this tissue were then cocultured with heat-inactivated hRSV-pulsed dendritic cells. The coculture was stimulated with heat-inactivated hRSV, and after the T cell expansion, the repertoire of TCRs expressed by T cells derived from the lung was evaluated by flow cytometry. The spleens of mice sacrificed at seventh day postinfection were collected, and the resulting splenocytes cells were then stimulated with heat-inactivated hRSV (HI-hRSV). The repertoire of the CD4^+^ (**A**) and CD8^+^ (**B**) in the spleen, and CD4^+^ (**C**) and CD8^+^ (**D**) in the lungs evaluated by flow cytometry. *N* experimental spleen mock = 11 and hRSV = 12. For the lungs, a pool of the lungs was performed, resulting in 3 for mock and 4 for hRSV. Expression of TCRVβ was compared between mock-treated and hRSV-infected mice. ANOVA unpaired test was performed to assess statistical differences. * *p* < 0.05. Bars represent mean ± SD.

The repertoire of the CD4^+^ T cells was found diverse in the spleen from both mock-treated and hRSV-infected mice (Figure 2A). Also, a significantly lower expression of TCRVβ7 and higher TCRVβ8.3 was observed for hRSV-infected as compared to mock-treated mice (Figure 2A). Moreover, the expression of TCRVβ2, 3, 4, 5.1,5.2, and 17 in CD8^+^ T cells was significantly higher in the hRSV-infected as compared to the mock-treated mice (Figure 2B). On the other hand, the expression of the TCRVβ6, 7, and 14 in CD8^+^ T cells was decreased for the hRSV-infected as compared to the mock-treated mice. Those differences could reveal characteristic TCRVβ usages in response to hRSV infection. 

To determine the repertoire of the TCR in the lungs, purified T cells from this tissue were then cocultured with heat-inactivated hRSV-pulsed DCs. Due to that only a few T cells could be obtained from one lung, T cells derived from three lungs of mice receiving the same treatment were pooled and further analyzed. The coculture was stimulated with heat-inactivated hRSV and after the in vitro expansion of lung-derived T cells the TCR repertoire was determined by flow cytometry. Three experimental groups showed a slight increase of CD69 expression after heat-inactivated hRSV stimulation, and higher CD69 expression in unstimulated cells from vaccinated mice compared to mock-treated and hRSV-infected mice was found (Figure 2D). It was observed that T cells expanded from the lungs from hRSV-infected mice presented no significant differences in the expression of the TCRVβ^+^ in the lungs as compared to the mock-treated mice (Figure 2C,D).

### 3.2. TCR Repertoire of T Cells from hRSV-Infected Mice Previously Immunized with rBCG-N-hRSV

**Figure 3:**
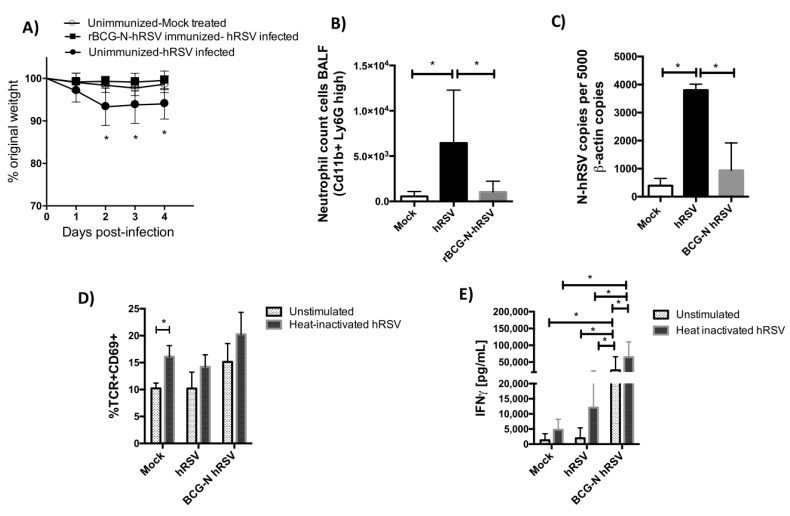
T cell expansion induced by hRSV infection in rBCG-N-hRSV vaccinated mice. BALB/cJ mice were immunized with BCG-N, and 14 days postimmunization were infected with 1 × 10^6^ PFU of hRSV. Four days after infection, mice were euthanized. Bodyweight was measured for four days (**A**). Also, neutrophils count (**B**) in the BALF was evaluated using FACS. (**C**) Viral loads were evaluated by RT-qPCR. (**D**) The spleens of mice sacrificed at seventh day postinfection were collected, and the resulting splenocytes were then stimulated with heat-inactivated hRSV (HI-hRSV) (57 °C for 30 min). Seventy-two hours poststimulation, TCR^+^ CD69^+^ cells as a T cell activation marker were detected by flow cytometry (d) and IFN-γ production was detected by ELISA (**E**). (**A**) Two-way ANOVA paired test and multiple *t*-students test were performed to assess statistical differences (* *p* < 0.05) indicating statistical difference in (i) time and infection/treatment condition, (ii) significant decrease of percent original weight in hRSV-infected as compared to mock-treated and to rBCG-N-hRSV-inmunized hRSV-infected mice at days 2, 3 and 4 postinfection. (**B**–**E**) *t*-students tests were performed (* *p* < 0.05). Bars represent mean ± SD in all graphs.

Next, we evaluated the TCR repertoire for T cells obtained from mice infected with hRSV that were either naïve or previously immunized with the rBCG-N-hRSV vaccine (Add Refs). Vaccinated mice received 1 x 10^8^ CFU of a recombinant BCG strain expressing the N protein of hRSV at days 1 and 14 of the experiment. Twenty-one days after the first immunization, mice were challenged with 1 × 10^6^ PFU of hRSV and euthanatized four days postinfection. For these experiments, mice were euthanized at day 4 because early recruitment of T cells was previously reported after rBCG-N-hRSV vaccination in mice [8]. As expected, a significant loss of original weight was shown by hRSV-infected mice compared to the mock-treated and immunized mice (Figure 3A). Infiltration of neutrophils was observed in the hRSV-infected mice, as compared to the mock-treated mice (Figure 3B). Importantly, the cell infiltration into the lungs was significantly reduced in the rBCG-N-hRSV-immunized mice, as compared to unimmunized hRSV-infected animals (Figure 3B). As previously reported, decreased of viral loads was found for the rBGC-N-hRSV-immunized mice (Figure 3C). After the expansion of hRSV-specific T cells in the spleen, activation of T cells (defined as CD69^+^ TCR^+^) after the stimulation with the heat-inactivated hRSV was observed (Figure 3D). The expansion of hRSV-specific T cells from the spleens of rBCG-N-hRSV-immunized and hRSV-infected mice led to activated T cells (TCR^+^CD69^+^) in these cultures. Furthermore, the specific-hRSV response induced by hRSV-infected rBCG-N-hRSV-immunized mice was supported by the secretion of IFN-γ by splenocytes stimulated with heat-inactivated hRSV, as compared to unstimulated or unimmunized controls. Also, significantly higher production of IFN-γ was observed for splenocytes derived from vaccinated mice stimulated with heat-inactivated hRSV as compared to mock-treated mice (Figure 3E). It has been previously shown that BCG vaccine per se induces a favorable Th1 immune response [28], which is consistent with the IFN-γ levels found in splenocytes without stimulation from rBCG-N-hRSV-immunized mice challenged with hRSV (Figure 3E). Importantly, we have already shown an hRSV-specific protective response of rBCG-N-hRSV immunization with prevention of body weight loss, reduction of immune cells infiltration into the lungs as compared to immunization with the wild type BCG vaccine [6,9].

**Figure 4:**
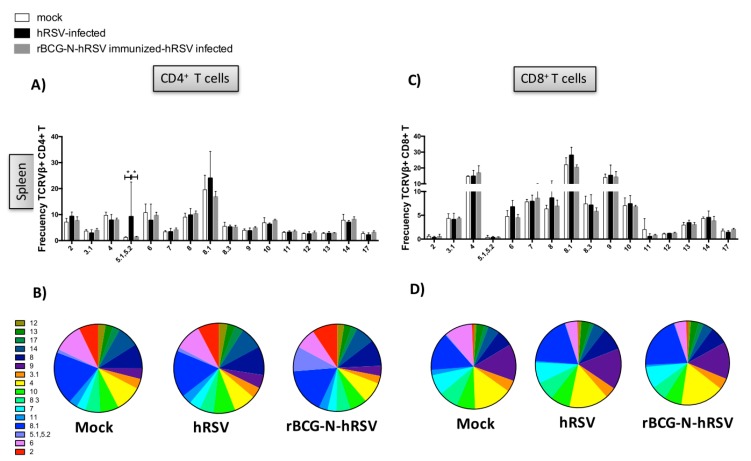
TCRVβ^+^ expression in the CD4^+^ and CD8^+^ T cells in spleen in the context of infection and immunization. BALB/cJ mice were immunized with rBCG-N-hRSV and 14 days postimmunization were infected with 1 × 10^6^ PFU of hRSV. Four days after infection, mice were euthanized. The spleens of mice sacrificed at seventh day postinfection were collected, and the resulting splenocytes were then stimulated with heat-inactivated hRSV (HI-hRSV) (57 °C for 30 min). Seventy-two days poststimulation, the expression of different TCRVβ+ was evaluated by flow cytometry in rBCG-N-hRSV immunized and hRSV-infected mice. The expression of different TCRVβ^+^ was evaluated by flow cytometry in rBCG-N-hRSV immunized and hRSV-infected mice in CD4^+^ and CD8^+^ T cells. Frequencies are shown in bars (**A**,**C**) and in pie charts (**B**,**D**). The differences between TCRVβ^+^ expression among the different experimental groups were determined using multiple *t*-students tests, * *p* < 0.05. Bars represent mean ± SD in all graphs.

**Figure 5:**
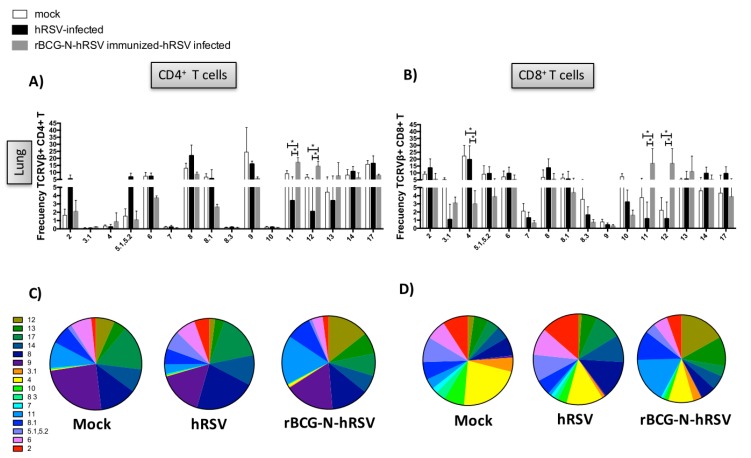
TCRVβ^+^ expression in the CD4^+^ and CD8^+^ T cells in the lungs in the context of infection and immunization. BALB/cJ mice were immunized with rBCG-N-hRSV and 14 days postimmunization were infected with 1 × 10^6^ PFU of hRSV. Four days after infection, mice were euthanized. The expression of different TCRVβ^+^ in CD4^+^ and CD8^+^ T cells was evaluated by flow cytometry in lungs extracted from rBCG-N-hRSV immunized and hRSV-infected mice. Frequencies are shown in bars (**A**,**C**) and in pie charts (**B**,**D**). The differences between TCRVβ^+^ expression among the different experimental groups were determined using multiple *t*-students tests, * *p* < 0.05. Bars represent mean ± SD in all graphs.

To determine whether the use of the TCR repertoire was modified after the immunization with the rBCG-N-hRSV vaccine, the expression of the different TCRVβ^+^ in CD4^+^ and CD8^+^ T cells in the lungs and spleens among the different experimental groups was evaluated (Figure 4 and Figure 5). In this case, the TCR repertoire analysis was performed stimulating splenocytes with HI-hRSV as described above. However, the TCR repertoire analysis for lungs was performed by determining the TCR expression without antigen stimulation to obtain the total T cells for each experimental group. Due that rBCG-N-hRSV-immunized mice showed a significant increase in T cell recruitment to the lungs as compared to unimmunized mice, the total count of all TCRVβ^+^ evaluated was included for the TCR repertoire analyses (Figure 4 and Figure 5). Then, to obtain the frequency of a certain TCRVβ, the following formula was used: (TCRVβ^+^ CD4^+^ or CD8^+^ T cell count / Sum of TCRVβ^+^ evaluated CD4^+^ or CD8^+^ T cell count) × 100. It is noteworthy that differences previously found in TCRVβ^+^ expression (TCRVβ^+^7 and TCRVβ^+^8.3) in spleen cells comparing mock-treated and hRSV-infected mice were not observed for these cells (immunized experimental set, Figure 4). This could be explained for the fact that this second set of experiments hRSV infection was performed 21 days postimmunization; herein the euthanization was done for ten-week-old mice instead of seven-week-old mice. The expression of TCRVβ^+^11 and TCRVβ^+^12 were significantly increased in the lungs from CD4^+^ and CD8^+^ T cells in rBCG-N-hRSV-immunized hRSV-infected mice as compared to nonimmunized animals (Figure 4 and Figure 5). Additionally, the expression of the TCRVβ^+^4 was significantly increased in the unimmunized hRSV-infected mice as compared to the mock and immunized with the rBCG-N-hRSV (Figure 4 and Figure 5). On the other hand, an increase of the expression of the TCRVβ^+^5.1,5.2 CD4^+^ T cells in spleens from unimmunized hRSV-infected mice was observed (Figure 5). These results suggest that rBCG-N-hRSV vaccination modified the TCR repertoire, which seemed particularly diversified in the lungs, suggesting an increased capacity to respond to a larger number of antigens. Regarding T cells, in the lungs from rBCG-N-hRSV-immunized hRSV-infected mice the TCRVβ^+^11 and 12 frequencies were significantly increased as compared to mock-treated and hRSV-infected mice. Importantly, those two dominant TCRVβ populations could be important in the protective role of rBCG-N-hRSV against hRSV infection (Figure 5).

As the T cell repertoire consists of different populations with certain TCRs, it is important to study the proportion of each TCR with a particular TCRVβ chain in the whole population for each experimental group of mice. Then, to determine the diversity of the TCRVβ^+^ repertoire in the site of the infection, the expression of each different TCRVβ^+^ by CD4^+^ and CD8^+^ T cells in the spleen and lungs for all experimental groups is shown as pie charts (Figure 4B,D and Figure 5B,D).

## 4. Discussion

The TCRVβ usage by CD4^+^ and CD8^+^ T cells expanded in response to hRSV infection has not been thoroughly characterized. Due to the critical role of T cells in clearing an acute hRSV infection as well as the importance of Th1/Th2 balance in the outcome of hRSV infection, it is important to better characterize the TCR diversity both in mice immunized with rBCG-N-hRSV and naïve mice infected with hRSV. Such a characterization of TCRVβ repertoire could provide new insights about the molecular features of T cell antigen receptors contributing to either the generation of lung immunopathology or to viral clearance, as well as for the resolution of disease as observed in rBCG-N-hRSV-immunized mice. TCRVβ repertoire differences between peripheral CD4^+^ and CD8^+^ T cell could lead to variant T cell avidities during anti-pathogen immune responses [29]. Consistently, we found differences in the TCRVβ repertoire for both CD4^+^ and CD8^+^ T cells during hRSV infection and rBCG-N-hRSV immunization, followed by hRSV infection. For both CD4^+^ and CD8^+^ T cells, the frequencies of TCRVβ11 and TCRVβ12 were increased, while TCRVβ4 and TCRVβ17 decreased in the lungs from rBCG-N-hRSV immunized and hRSV-infected as compared to mock-treated mice. Herein, those TCRVβ^+^ could be responsible for the hRSV pathology in naïve mice or the protective response induced by immunization with the rBCG-N-hRSV vaccine [6,8,9]. Along these lines, it remains to be elucidated whether such contribution is different whether it involves the resolution of infection or the prevention of a secondary exposure to hRSV.

T cell expansion from spleen and lungs of hRSV-infected and rBCG-N-hRSV-immunized mice was performed in the present work. Although CD69 expression was found after stimulation with heat-inactivated hRSV in all experimental groups including mock-treated mice, enough T cells were obtained to measure the TCR repertoire and differences in TCRVβ expression between groups. Also, some variation on TCRVβ expression in spleen cells was observed between unimmunized and immunized experimental sets (Figure 2 and Figure 4), which could be explained by age differences between mice. In particular, hRSV-specific T cells in cultures from mock-treated might be low due to the short protocol of antigenic stimulation performed in this work. As previously described, TCRVβ8.1 was predominant in CD4^+^ T cells expanded from spleens of hRSV-infected mice. Interestingly, TCRVβ5.1,5.2 was significantly higher in CD4^+^ T cells expanded from the spleens of hRSV-infected mice as compared to mock-treated and rBCG-N-hRSV immunized mice, showing that these latter groups had similar frequencies. Those differences in specific TCRVβ populations could be involved in the pathogenicity of hRSV or the protection of rBCG-N-hRSV vaccine. Hence, the study of the TCR repertoire in response to hRSV infection provides information about the T cell response to all proteins of the virus, including those epitopes that are considered subdominant epitopes and that could also be important for the immune response to hRSV. Moreover, the distribution of T cells into the tissues, including spleen and the lungs, may affect the frequencies of epitope-specific T cells [30], thereby being important to evaluate the TCR repertoire in the context of hRSV natural infection.

T cells expanded from the spleen with hRSV stimulus showed a more diverse TCRVβ repertoire as compared to T cells derived from the lungs. Whether a limited selection of TCR has an impact on the antiviral response of the host and the resolution of the disease remains to be determined. An explanation that has been proposed is that some TCRs may help to control pathogens since the virus may have less probability to escape via antigenic variation [25]. In noninfectious diseases, such as monoclonal human lymphomas and lymphoid leukemias, TCRVβ rearrangements are used as highly sensitive markers for diagnosis and follow-up of the disease [31,32]. Further, non-Hodgkin’s lymphomas patients have been shown to express the same TCRVβ subfamilies as compared to healthy controls [31]. Alternatively, limited TCR selection has also been shown as a consequence of chronic exposure to antigens, such as viral infections including hepatitis B, cytomegalovirus, influenza and human immunodeficiency virus (HIV) [33,34,35]. Thus, the TCR repertoire in these cases is important for the diagnosis and follow-up of these diseases.

Interestingly, it has been shown that the TCR repertoire is narrowed after secondary exposure to a virus [36]. However, this limited TCR selection does not necessarily require chronic exposure to a given antigen such as in hRSV infection. Along these lines, the rBCG-N-hRSV vaccine exhibited higher TCR diversity in CD4^+^ T cells from the lungs, as compared to the other experimental groups, suggesting that mice immunized with the rBCG-N-hRSV vaccine can respond to a more diverse array of antigens and with different T helper capacities. Importantly, the better understanding of phenotype of those CD4^+^ T cell populations, such as their polarizing ability and cytokine profile, would be an important contribution to discerning what is required for a protective T cell immunity to the virus. Furthermore, as hRSV infects the lung, such TCR^+^ clones found in rBCG-N-hRSV-immunized mice could be lung-resident memory T cells, important cells for protecting against respiratory pathogens [37,38]. Consistently, it has been shown that BCG-induced granuloma T cells express a diverse TCR repertoire, but single specificity of CD4^+^ T cells is required to form the granuloma and control the BCG infection [39]. Therefore, the question as to whether those T cells found in rBCG-N-hRSV-immunized mice are all hRSV-specific and able to protect against hRSV infection remains to be answered.

Our data provide additional insights as to how the rBCG-N-hRSV not only can prevent the hRSV-caused disease, as previously described [9], but also this vaccine can shape the TCR repertoire for the T cells expanded by hRSV infection. A variant TCRVβ repertoire was found in unimmunized and immunized-hRSV infected mice. Also, T cells expanded from the spleen of hRSV-infected mice displayed a more diverse TCR repertoire as compared to T cells found in the lungs. Such results underscore the relevance of the TCR repertoire for the outcome of the hRSV-associated disease.

## Supplementary Materials

The following are available online at https://www.mdpi.com/1999-4915/12/2/233/s1, Figure S1: Gating strategy to determine the frequency of each TCRVβ for hRSV-induced T cells.

## References

[1] Nair H., Nokes D.J., Gessner B.D., Dherani M., Madhi S.A., Singleton R.J., O’Brien K.L., Roca A., Wright P.F., Bruce N. (2010). Global burden of acute lower respiratory infections due to respiratory syncytial virus in young children: A systematic review and meta-analysis. Lancet.

[2] Bendelja K., Gagro A., Bace A., Lokar-Kolbas R., Krsulovic-Hresic V., Drazenovic V., Mlinaric-Galinovic G., Rabatic S. (2000). Predominant type-2 response in infants with respiratory syncytial virus (rsv) infection demonstrated by cytokine flow cytometry. Clin. Exp. Immunol..

[3] Bertrand P., Lay M.K., Piedimonte G., Brockmann P.E., Palavecino C.E., Hernandez J., Leon M.A., Kalergis A.M., Bueno S.M. (2015). Elevated il-3 and il-12p40 levels in the lower airway of infants with rsv-induced bronchiolitis correlate with recurrent wheezing. Cytokine.

[4] Graham B.S., Perkins M.D., Wright P.F., Karzon D.T. (1988). Primary respiratory syncytial virus infection in mice. J. Med. Virol..

[5] Kong X., Hellermann G.R., Patton G., Kumar M., Behera A., Randall T.S., Zhang J., Lockey R.F., Mohapatra S.S. (2005). An immunocompromised balb/c mouse model for respiratory syncytial virus infection. Virol. J..

[6] Bueno S.M., Gonzalez P.A., Cautivo K.M., Mora J.E., Leiva E.D., Tobar H.E. (2008). Protective t cell immunity against respiratory syncytial virus is efficiently induced by recombinant bcg. Proc. Natl. Acad. Sci. USA.

[7] Graham B.S., Bunton L.A., Wright P.F., Karzon D.T. (1991). Role of t lymphocyte subsets in the pathogenesis of primary infection and rechallenge with respiratory syncytial virus in mice. J. Clin. Investig..

[8] Cautivo K.M., Bueno S.M., Cortes C.M., Wozniak A., Riedel C.A., Kalergis A.M. (2010). Efficient lung recruitment of respiratory syncytial virusspecific th1 cells induced by recombinant bacillus calmette-guerin promotes virus clearance and protects from infection. J. Immunol..

[9] Céspedes P.C., Rey-Jurado E., Espinoza J.A., Rivera C.A., Canedo-Marroquín G., Bueno S.M., Kalergis A. (2017). A single, low dose of a cgmp recombinant bcg vaccine elicits protective t cell immunity against the human respiratory syncytial virus infection and prevents lung pathology in mice. Vaccine.

[10] Hodges E., Krishna M.T., Pickard C., Smith J.L. (2003). Diagnostic role of tests for t cell receptor (tcr) genes. J. Clin. Pathol..

[11] Arstila T.P., Casrouge A., Baron V., Even J., Kanellopoulos J., Kourilsky P. (1999). A direct estimate of the human alphabeta t cell receptor diversity. Science.

[12] Murphy J., Summer R., Wilson A.A., Kotton D.N., Fine A. (2008). The prolonged life-span of alveolar macrophages. Am. J. Respir. Cell Mol. Biol..

[13] Blum J.S., Wearsch P.A., Cresswell P. (2013). Pathways of antigen processing. Annu. Rev. Immunol..

[14] Chang J., Srikiatkhachorn A., Braciale T.J. (2001). Visualization and characterization of respiratory syncytial virus f-specific cd8(+) t cells during experimental virus infection. J. Immunol..

[15] Jiang S., Borthwick N.J., Morrison P., Gao G.F., Steward M.W. (2002). Virus-specific ctl responses induced by an h-2k(d)-restricted, motif-negative 15-mer peptide from the fusion protein of respiratory syncytial virus. J. Gen. Virol..

[16] Johnstone C., de León P., Medina F., Melero J.A., García-Barreno B., Del Val M. (2004). Shifting immunodominance pattern of two cytotoxic t-lymphocyte epitopes in the f glycoprotein of the long strain of respiratory syncytial virus. J. Gen. Virol..

[17] Kulkarni A.B., Collins P.L., Bacik I., Yewdell J.W., Bennink J.R., Crowe J.E., Murphy B.R. (1995). Cytotoxic t cells specific for a single peptide on the m2 protein of respiratory syncytial virus are the sole mediators of resistance induced by immunization with m2 encoded by a recombinant vaccinia virus. J. Virol..

[18] Gil A., Yassai M.B., Naumov Y.N., Selin L.K. (2015). Narrowing of human influenza a virus-specific t cell receptor α and β repertoires with increasing age. J. Virol..

[19] Miles J.J., Thammanichanond D., Moneer S., Nivarthi U.K., Kjer-Nielsen L., Tracy S.L., Aitken C.K., Brennan R.M., Zeng W., Marquart L. (2011). Antigen-driven patterns of tcr bias are shared across diverse outcomes of human hepatitis c virus infection. J. Immunol..

[20] Hernandez D.M., Valderrama S., Gualtero S., Hernandez C., Lopez M., Herrera M.V., Solano J., Fiorentino S., Quijano S. (2018). Loss of t-cell multifunctionality and tcr-vbeta repertoire against epstein-barr virus is associated with worse prognosis and clinical parameters in hiv(+) patients. Front. Immunol..

[21] de Vos van Steenwijk P.J., Heusinkveld M., Ramwadhdoebe T.H., Lowik M.J., van der Hulst J.M., Goedemans R., Piersma S.J., Kenter G.G., van der Burg S.H. (2010). An unexpectedly large polyclonal repertoire of hpv-specific t cells is poised for action in patients with cervical cancer. Cancer Res..

[22] Varga S.M., Wang X., Welsh R.M., Braciale T.J. (2001). Immunopathology in rsv infection is mediated by a discrete oligoclonal subset of antigen-specific cd4(+) t cells. Immunity.

[23] Bar-Haim E., Erez N., Malloy A.M., Graham B.S., Ruckwardt T.J. (2014). Cd8+ tcr transgenic strains expressing public versus private tcr targeting the respiratory syncytial virus k(d)m2(82-90) epitope demonstrate similar functional profiles. PLoS ONE.

[24] Vallbracht S., Jessen B., Mrusek S., Enders A., Collins P.L., Ehl S., Krempl C.D. (2007). Influence of a single viral epitope on t cell response and disease after infection of mice with respiratory syncytial virus. J. Immunol..

[25] Cornberg M., Chen A.T., Wilkinson L.A., Brehm M.A., Kim S.K., Calcagno C., Ghersi D., Puzone R., Celada F., Welsh R.M. (2006). Narrowed tcr repertoire and viral escape as a consequence of heterologous immunity. J. Clin. Investig..

[26] Soto J.A., Galvez N., Rivera C.A., Palavecino C.E., Céspedes P.F., Rey-Jurado E., Bueno S.M., Kalergis A.M. (2018). Recombinant bcg vaccines reduce pneumovirus-caused airway pathology by inducing protective cellular and humoral immunity rbcg induces protection against pneumoviruses. Submitt. Front. Immunol..

[27] Herrada A.A., Contreras F.J., Tobar J.A., Pacheco R., Kalergis A.M. (2007). Immune complex-induced enhancement of bacterial antigen presentation requires fcγ receptor iii expression on dendritic cells. Proc. Natl. Acad. Sci. USA.

[28] Marchant A., Goetghebuer T., Ota M.O., Wolfe I., Ceesay S.J., De Groote D., Corrah T., Bennett S., Wheeler J., Huygen K. (1999). Newborns develop a th1-type immune response to mycobacterium bovis bacillus calmette-guerin vaccination. J. Immunol..

[29] Nakatsugawa M., Rahman M.A., Yamashita Y., Ochi T., Wnuk P., Tanaka S., Chamoto K., Kagoya Y., Saso K., Guo T. (2016). Cd4(+) and cd8(+) tcrβ repertoires possess different potentials to generate extraordinarily high-avidity t cells. Sci. Rep..

[30] Lee S., Miller S.A., Wright D.W., Rock M.T., Crowe J.E. (2007). Tissue-specific regulation of cd8+ t-lymphocyte immunodominance in respiratory syncytial virus infection. J. Virol..

[31] Fozza C., Corda G., Virdis P., Contini S., Barraqueddu F., Galleu A., Isoni A., Cossu A., Dore F., Careddu M.G. (2015). Derangement of the t-cell repertoire in patients with b-cell non-hodgkin’s lymphoma. Eur. J. Haematol..

[32] Salameire D., Solly F., Fabre B., Lefebvre C., Chauvet M., Gressin R., Corront B., Ciapa A., Pernollet M., Plumas J. (2012). Accurate detection of the tumor clone in peripheral t-cell lymphoma biopsies by flow cytometric analysis of tcr-vβ repertoire. Mod. Pathol..

[33] Wang G.C., Dash P., McCullers J.A., Doherty P.C., Thomas P.G. (2012). T cell receptor αβ diversity inversely correlates with pathogen-specific antibody levels in human cytomegalovirus infection. Sci. Transl. Med..

[34] Meyer-Olson D., Shoukry N.H., Brady K.W., Kim H., Olson D.P., Hartman K., Shintani A.K., Walker C.M., Kalams S.A. (2004). Limited t cell receptor diversity of hcv-specific t cell responses is associated with ctl escape. J. Exp. Med..

[35] Costa A., Koning D., Ladell K., McLaren J.E., Grady B.P., Schellens I.M., Ham P.v., Nijhuis M., Borghans J.A., Keşmir C. (2015). Complex t-cell receptor repertoire dynamics underlie the cd8+ t-cell response to hiv-1. J. Virol..

[36] Zhong W., Reinherz E.L. (2004). In vivo selection of a tcr vβ repertoire directed against an immunodominant influenza virus ctl epitope. Int. Immunol..

[37] Pizzolla A., Nguyen T.H., Sant S., Jaffar J., Loudovaris T., Mannering S.I., Thomas P.G., Westall G.P., Kedzierska K., Wakim L.M. (2018). Influenza-specific lung-resident memory t cells are proliferative and polyfunctional and maintain diverse tcr profiles. J. Clin. Investig..

[38] Retamal-Diaz A., Covian C., Pacheco G.A., Castiglione-Matamala A.T., Bueno S.M., Gonzalez P.A., Kalergis A.M. (2019). Contribution of resident memory cd8(+) t cells to protective immunity against respiratory syncytial virus and their impact on vaccine design. Pathogens.

[39] Hogan L.H., Macvilay K., Barger B., Co D., Malkovska I., Fennelly G., Sandor M. (2001). Mycobacterium bovis strain bacillus calmette-guerin-induced liver granulomas contain a diverse tcr repertoire, but a monoclonal t cell population is sufficient for protective granuloma formation. J. Immunol..

