# Supplementary material for: TCR Repertoire Characterization for T Cells Expanded in Response to hRSV Infection in Mice Immunized with a Recombinant BCG Vaccine
Source: Viruses. 2020 Feb 20;12(2):233. doi: 10.3390/v12020233 (PMC7077260; doi:10.3390/v12020233)
Supplement: Supplementary file 1 [file viruses-12-00233-s001.pdf]

Supplementary Figure 1

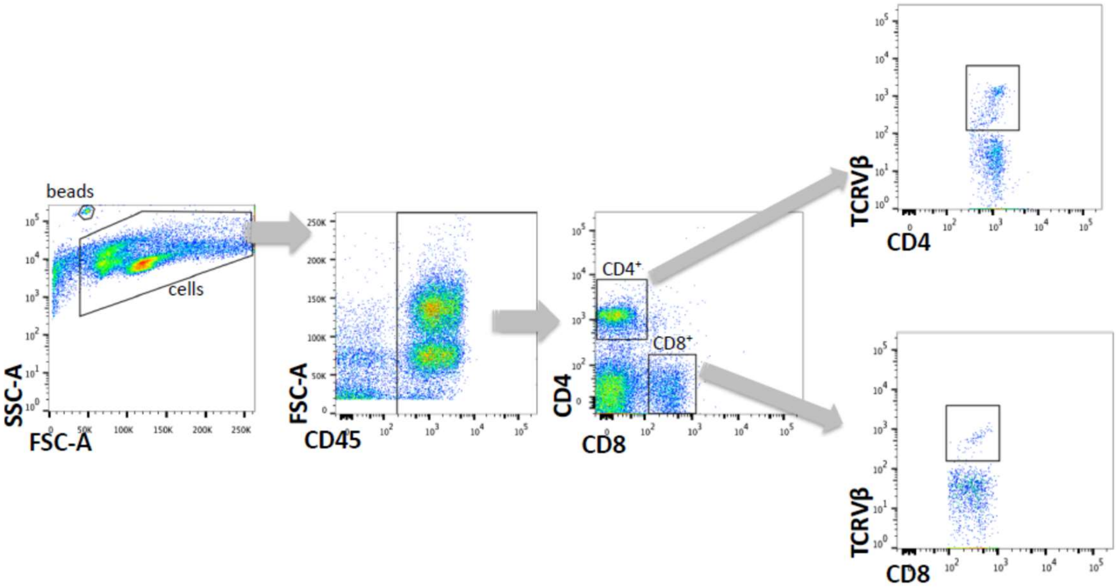

Figure S1. Gating strategy to determine the frequency of each TCRVβ for hRSV-induced T cells. Spleen and lungs from infected mice were stained and evaluated by flow cytometry. Dot plots of gating strategy are shown. CD45⁺ cells were gated and then the positive cells for CD4⁺ and CD8⁺ T cells were selected. Finally, over the CD4⁺ and CD8⁺ T cells gating, we selected the positive cells for each respective TCRVβ.
